# Correlations between DW‐MRI and ^18^F‐FDG PET/CT parameters in head and neck squamous cell carcinoma following definitive chemo‐radiotherapy

**DOI:** 10.1002/cnr2.1360

**Published:** 2021-05-07

**Authors:** Steve Connor, Cherry Sit, Mustafa Anjari, Teresa Szyszko, Joel Dunn, Irumee Pai, Gary Cook, Vicky Goh

**Affiliations:** ^1^ School of Biomedical Engineering and Imaging Sciences St Thomas' Hospital, King's College London UK; ^2^ Department of Neuroradiology King's College Hospital NHS Foundation Trust London UK; ^3^ Department of Radiology Guy's and St Thomas' NHS Foundation Trust London UK; ^4^ King's College London & Guy's and St. Thomas' PET Centre London UK; ^5^ Department of Otolaryngology Guy's and St Thomas' NHS Foundation Trust London UK

**Keywords:** carcinoma, squamous cell, chemo‐radiotherapy, diffusion‐weighted magnetic resonance imaging, head and neck cancer, positron emission tomography, posttreatment

## Abstract

**Background:**

Posttreatment diffusion–weighted magnetic resonance imaging (DW‐MRI) and 18F‐fluorodeoxygluocose (^18^F‐FDG) positron emission tomography (PET) with computed tomography (PET/CT) have potential prognostic value following chemo‐radiotherapy (CRT) for head and neck squamous cell carcinoma (HNSCC). Correlations between these PET/CT (standardized uptake value or SUV) and DW‐MRI (apparent diffusion coefficient or ADC) parameters have only been previously explored in the pretreatment setting.

**Aim:**

To evaluate stage III and IV HNSCC at 12‐weeks post‐CRT for the correlation between SUV_max_ and ADC values and their interval changes from pretreatment imaging.

**Methods:**

Fifty‐six patients (45 male, 11 female, mean age 59.9 + − 7.38) with stage 3 and 4 HNSCC patients underwent 12‐week posttreatment DW‐MRI and ^18^F‐FDG PET/CT studies in this prospective study. There were 41/56 patients in the cohort with human papilloma virus‐related oropharyngeal cancer (HPV OPC). DW‐MRI (ADC_max_ and ADC_min)_ and ^18^F‐FDG PET/CT (SUV_max_ and SUV_max_ ratio to liver) parameters were measured at the site of primary tumors (n = 48) and the largest lymph nodes (n = 52). Kendall's tau evaluated the correlation between DW‐MRI and ^18^F‐FDG PET/CT parameters. Mann‐Whitney test compared the post‐CRT PET/CT and DW‐MRI parameters between those participants with and without 2‐year disease‐free survival (DFS).

**Results:**

There was no correlation between DW‐MRI and ^18^F‐FDG PET/CT parameters on 12‐week posttreatment imaging (*P* = .455‐.794; tau = −0.075‐0.25) or their interval changes from pretreatment to 12‐week posttreatment imaging (*P* = .1‐.946; tau = −0.194‐0.044). The primary tumor ADC_mean_ (*P* = .03) and the interval change in nodal ADC_min_ (*P* = .05) predicted 2‐year DFS but none of the ^18^F‐FDG PET/CT parameters were associated with 2‐year DFS.

**Conclusions:**

There is no correlation between the quantitative DWI‐MRI and ^18^F‐FDG PET/CT parameters derived from 12‐week post‐CRT studies. These parameters may be independent biomarkers however in this HPV OPC dominant cohort, only selected ADC parameters demonstrated prognostic significance.

Study was prospectively registered at http://www.controlled-trials.com/ISRCTN58327080

## 1 BACKGROUND

Head and neck squamous cell carcinoma (HNSCC) is the seventh most common cancer worldwide.^1^ Patients with advanced loco‐regional disease can be treated with radiotherapy, or combined chemo‐radiotherapy (CRT), but 25% to 50% will have residual disease that requires further intervention.^2^, ^3^ Early posttreatment detection of residual tumor is required in order to optimize the outcomes of salvage surgery.^4^, ^5^ Unfortunately, clinical assessment and structural imaging are limited in their ability to detect loco‐regional residual or recurrent disease due to treatment‐related soft tissue distortion.^6^, ^7^, ^8^

In order to overcome the shortcomings of structural imaging in this clinical scenario, metabolic imaging with 18F‐fluorodeoxygluocose (^18^F‐FDG) positron emission tomography (PET) in combination with computed tomography (PET/CT) has become widely utilized.^2^, ^9^, ^10^, ^11^, ^12^ ^18^F‐FDG PET/CT is reported to be a highly sensitive technique for detection of HNSCC in the post‐CRT setting. Semiquantitative analysis of maximum standardized uptake value (SUV_max_) with ^18^F‐FDG PET‐CT has been shown to have prognostic significance, with increased SUV_max_ being associated with treatment failure.^13^, ^14^, ^15^, ^16^, ^17^, ^18^

Quantitative diffusion‐weighted magnetic resonance imaging (DW‐MRI) with measurement of the apparent diffusion coefficient (ADC) is another functional imaging technique, which may be used to help distinguish tumor from posttreatment changes and has been applied to the early posttreatment assessment of HNSCC. The majority of studies have found that an increased posttreatment ADC or greater rise in ADC from the pretreatment to the intratreatment or posttreatment studies is a predictor of treatment success.^19^, ^20^, ^21^, ^22^, ^23^, ^24^

It is still unclear whether ^18^F‐FDG and ADC values provide similar information with regard to viable tumor cells, or whether the two are entirely unrelated biomarkers. Both ADC values and SUV_max_ have shown significant correlation with different histopathological parameters although this may depend on tumor grade.^25^, ^26^, ^27^, ^28^ There is currently no comparative data on the ability of post‐CRT quantitative ^18^F‐FDG PET/CT and DW‐MRI to predict post‐CRT residual disease, with current evaluations having been limited to comparing these parameters in the pretreatment and intratreatment settings.^29^, ^30^, ^31^, ^32^, ^33^, ^34^, ^35^, ^36^, ^37^ On the one hand, since a post‐CRT reduction in SUV_max_ and interval increase in ADC are both markers of treatment success, it would seem logical to expect them to negatively correlate. On the other hand, they reflect different biological processes, so the possibility of independent biomarkers, which are complementary in stratifying the probability of residual disease, should also be explored.

Our hypothesis was that there would be a significant negative linear relationship between ADC and SUV values on 12‐week post‐CRT studies in patients with stage III and IV HNSCC. Thus, our primary objective was to determine any correlation between ADC and SUV_max_ values on 12‐week post‐CRT studies, and between their interval changes from pretreatment to 12‐week post‐CRT studies. Our secondary objective was to evaluate these posttreatment ADC and SUV_max_ values and their interval changes for their ability to predict 2‐year DFS outcomes.

## 2 METHOD

### 2.1 Participants

Participants were recruited for a prospective single center cohort observational study (http://www.controlled-trials.com/ISRCTN58327080) following Research Ethics Committee approval (REC reference 13/LO/1876) and informed consent.

Participants were eligible if: (a) there was a histologically confirmed stage III and IV primary SCC of the head and neck without distant metastatic disease, (b) a 1‐cm^2^ area of measurable primary tumor and/or nodal tumor on the basis of standard clinico‐radiological staging, and (c) curative CRT was planned. Exclusion criteria included prior CRT, an Eastern Cooperative Oncology Group (ECOG) performance status >2, inability to provide informed consent, known allergy to gadolinium‐based contrast medium and eGFR<30 mL/min.

### 2.2 Treatment and HPV status

Intensity‐modulated radiotherapy (IMRT) was delivered as per the standard of care which was 7‐Gy in 35 fractions; 2Gy per fraction delivered once daily, 5 days a week. Concomitant intravenous cisplatin at a dose of 35 mg/m^2^ every 7 days, starting on day 1 of radiotherapy, was used for all patients with adequate GFR and no contraindications to cisplatin (n = 47) with carboplatin being used if measured GFR < 50 or if patient had a history of hearing impairment (n = 16). Two patients received radiotherapy alone. HPV status was analyzed for all oropharyngeal and 1/13 other cancers. This involved p16 testing using an immune‐stain or high‐risk HPV DNA testing using in situ hybridization.

### 2.3 Imaging

Participants underwent MRI prior to commencement of CRT, while MRI and ^18^F‐FDG PET/CT were performed at 12 weeks after completion of treatment. Although pretreatment ^18^F‐FDG PET/CT imaging was not mandated as a component of the study, it was performed in selected patients according to the institutional protocol.

### 2.4 Magnetic resonance imaging

#### 2.4.1 Protocol and technique

Participants underwent full standard institutional head and neck soft tissue protocol MRI on a 1.5 Tesla Siemens Magnetom Aera system (Siemen Medical Systems GmbH, Erlangen, Germany) using a surface‐phased array neck coil (Table 1). In addition, an echo planar diffusion‐weighted sequence was acquired including matched images in the axial plane with multiple b‐values (0, 50, 100, 800, and 1500 s/mm^2^) and the following scan parameters: repetition time 5900 ms, echo time 60 ms, two signal averages, FOV 240 mm × 240 mm, slice thickness 4 mm with a 0.5 mm slice gap. ADC maps were calculated from the *b* = 100 and *b* = 800 values.

**TABLE 1:** MRI protocol

	Plane	Slice thickness/gap	TR/TE	Field of view	Number of averages	Pixel Bandwidth	Flip angle	Acquisition matrix
T1w	Axial	4/0	549/11	220 × 220	1	200	160	384/269
T2w	Axial	4/0	5830/102	220 × 220	1	190	150	384/346
T1w fat saturated‐DIXON postgadolinium	Axial	4/0	566/11	220 × 220	1	330	145	320 × 224
STIR	Coronal	3/0.3	3000/35 TI 140	260 × 260	1	220	160	320 × 224
T1w fat sat‐DIXON postgadolinium	Coronal	3/0.3	708/10	280 × 280	1	340	145	320 × 320

#### 2.4.2 MRI processing and analysis

Processing and analysis of diffusion imaging was performed offline. Data from our institutional Picture Archiving and Communication System (PACS) database were transferred to OsiriX v8.0.2, open‐source Mac‐based medical image processing software. The regions of interest (ROIs) were placed by a radiologist with 3 years of experience under the supervision of a radiologist with 21 years of experience, who provided training in five cases and ongoing review of a further five cases. Free hand ROIs were placed on the assessable primary tumor and/or largest lymph node using the OsiriX Draw tool with the images magnified to a standard 300%. They were defined on the diffusion‐weighted imaging (DWI) *b* = 800 map as a focus of increased signal relative to background, but with access to the other MRI sequences. Areas of necrosis (non‐enhancement and high B0 map signal) and peri‐tumoral oedema (avid enhancement and high B0 map signal) were avoided.

ROIs were placed on the baseline and 12‐week posttreatment MRI studies in sequence. If there was no longer a 6 mm short axis focus of DWI signal on the posttreatment studies at the location of the initial lesion, a standardized 6 mm diameter circular ROI was placed at its original location by reference to the other sequences and these ROIs were termed “nonmeasurable.” All ROIs were then translated directly to a calculated ADC map generated from the b100 and b800 images using the OsiriX ADCmap v1.9 plugin (https://web.stanford.edu/~bah/software/ADCmap/). ADC_mean_ and SD were recorded with ADC_min_ calculated as ADC_mean_ − one SD (rather than absolute ADC_min_).

### 2.5 ^18^F‐FDG PET/CT

#### 2.5.1 Protocol and technique

The ^18^F‐FDG PET/CT was performed as per institutional clinical practice. Participants were fasted for at least 6 hours prior to administration of 350‐400 MBq ^18^F‐FDG. PET/CT scans were acquired 90 minutes after injection from the upper thigh to the base of skull (arms up) with additional local views of the head and neck (arms down) on one of two PET/CT scanners (Siemens mCT Flow VST or GE Discovery DST 710). Images were acquired in 3D time‐of‐flight (TOF) acquisitions, according to local protocols. A low‐dose CT scan (140 kV, 10 mA, 0.5 second rotation time, and 40 mm collimation) was performed at the start in order to provide attenuation correction. Images were reconstructed using ordered subset expectation maximization (OSEM) method. For GE Discovery 710, the parameters were: Algorithm: “VPFX”, OSEM, time‐of‐flight, 2 iterations 24 subsets; Matrix size: 256 × 256 × 47; Pixel Spacing: 2.73 × 2.73 × 3.27; Post‐filter: Gaussian 6.4 mm FWHM. For the Siemens mCT Flow, the parameters were: Algorithm: OSEM, time‐of‐flight, 2 iterations 21 subsets; Matrix size: 200 × 200 × 46; Pixel Spacing 3.07 × 4.07 × 2; Post‐filter: Gaussian 5.0 mm FWHM.

#### 2.5.2 ^18^F‐FDG PET/CT data processing and analysis

On pretreatment ^18^F‐FDG PET/CT imaging, a 6 mm diameter volume of interest (VOI) was placed at the site of most intense FDG uptake at the site of either the primary lesion and/or the largest lymph node, which was matched to the ROI placed for the MRI analysis. The SUV_max_ was calculated with semiautomated software on a Hermes workstation (Hermes Gold 3, Stockholm). The VOIs were placed by a radiologist with 3 years' experience, under the supervision of a radiologist with 16 years' experience who provided training in five cases and review of further five cases.

The same method for VOI measurement was applied on 12‐week posttreatment ^18^F‐FDG PET‐CT imaging and SUV_max_ was recorded. The VOIs were chosen with MRI guidance, and always correlated with areas of increased tracer uptake on the pretreatment images. If there was reduced or no uptake on the posttreatment images relative to background, the posttreatment MRI images were referenced and a 6 mm VOI was placed at the same site as the MRI ROI. If necrosis was identified within a lesion, the area of necrosis was excluded as much as possible, and VOI was placed in the area of most intense tracer uptake. Areas of normal physiological uptake were avoided.

A freehand region of interest (ROI) was placed over the right lobe of the liver, at approximately its largest diameter, to record background liver SUV_max._ ^31^ This was performed in order to calculate the SUV_max_ ratio to liver parameter.

### 2.6 Treatment outcome

Outcome evaluation comprised clinical assessment at 1 year and 2 years following completion of treatment. A 12‐week posttreatment positron emission tomography computed tomography (PET CT) study was standard of care and was used to guide clinical management. Treatment failure was determined by cytological or histological confirmation (biopsy or resection) or by serial progression on imaging follow‐up. The 2‐year DFS was recorded according to status at 2 years following completion of treatment.

### 2.7 Statistical analysis

The Shapiro‐Wilks normality test determined a proportion of the ADC_mean_ and liver SUV_max_ data to show a significant deviation from normal (with a multiple comparison corrected threshold). Therefore, the primary descriptive statistics focused on the nonparametric median ± interquartile range) and the primary correlation was performed with the nonparametric Kendall's Tau method.

The correlation between ADC_mean_ and SUV_max_ at the tumor and nodal sites on the 12‐week post‐CRT studies, and the interval change from pretreatment imaging to 12‐week posttreatment studies was analyzed. The threshold for statistical significance was set at *P* < .05. This study provided 95% power to detect a true “moderate” correlation of tau = 0.34 at this threshold.

The same statistical approach was used to extend the comparison to alternative parameters (ADC_min_, SUV_max_ ratio to liver) on the 12‐week posttreatment studies, as well as the interval change from pretreatment imaging to 12‐week posttreatment studies. The subgroup of patients with measurable disease as defined by a clear focus of DWI signal on the 12‐week posttreatment DW‐MRI was also analyzed separately.

Scatter plots of ^18^F‐FDG PET/CT and MRI measures were produced to demonstrate any correlation with 95% confidence intervals.

The Mann‐Whitney test was used for a univariate analysis comparing tumor and nodal ADC and SUV_max_ parameters with the dichotomized 2‐year DFS.

## 3 RESULTS

### 3.1 Participants

**FIGURE 1:**
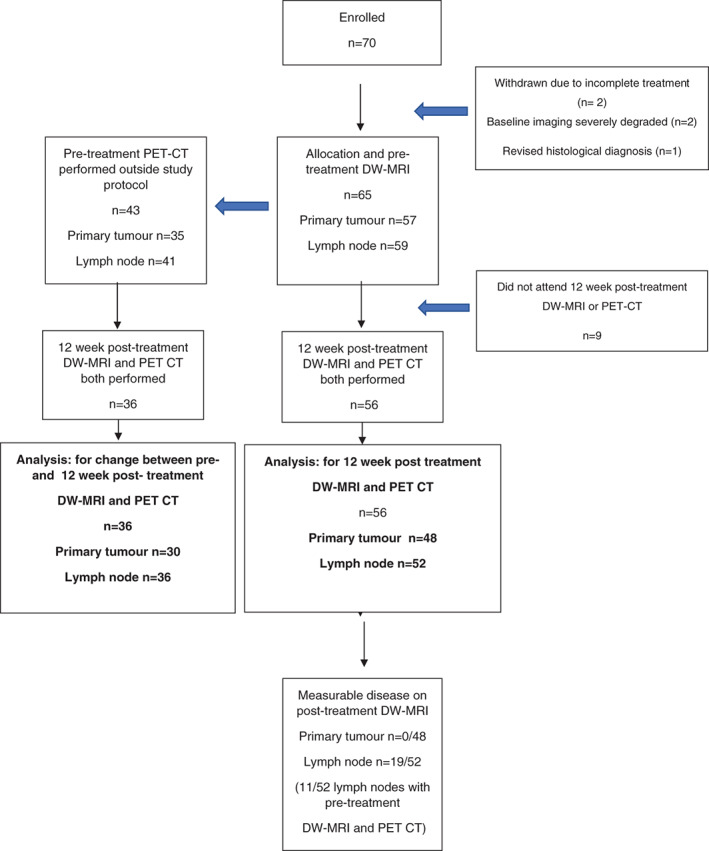
Participant flow chart

The participant flowchart is summarized in Figure 1.

There were 70 participants initially enrolled in the study. However, five patients were subsequently withdrawn and a further nine participants did not attend for one of the posttreatment studies (Figure 1). Therefore, 12‐week post‐CRT ^18^F‐FDG PET‐CT and DW‐MRI were analyzed for 56 patients (45 male, 11 female, mean age 59.9 + −7.38). Since the ROIs were only placed on 1 cm^2^ areas of measurable primary tumor and/or nodal tumor, the measurements were performed at the primary site alone (no measurable pathological lymph nodes, n = 4), the largest lymph node alone (no measurable primary tumor, n = 8) or both sites (n = 44). The tumor site, subsite, and HPV status are documented in Table 2. There was a majority of human papilloma virus‐associated oropharyngeal cancer (HPV OPC) participants in this prospectively recruited cohort.

**TABLE 2:** Patient characteristics of 56 patients (45 male, 11 female, mean age 59.9 + −7.38) patients, indicating site, subsites, and HPV status

**Oropharynx**	**n = 43**		
Tongue base	26	HPV +ve	41
Tonsil	16	HPV −ve	2
Soft Palate	1		
**Larynx**	**n = 8**		
Supraglottic	6	HPV +ve	0
Transglottic	2	HPV −ve	1
		Not tested	7
**Hypopharynx**	**n = 5**		
Piriform Fossa	5	HPV +ve	0
		HPV −ve	0
		Not tested	5

Since pretreatment ^18^F‐FDG PET/CT was performed according to the institutional protocol rather than under the research protocol, it was only available for 43 patients. There were 36/43 patients who also had 12‐week post‐CRT ^18^F‐FDG PET‐CT and DW‐MRI available. Therefore, the interval changes from pretreatment to 12‐week posttreatment studies could be analyzed in 36 patients with ROIs placed at the largest lymph node alone (n = 6) or both sites (n = 30).

At 2‐year follow‐up, there were three participants with isolated nodal recurrence, one participant with isolated primary recurrence and two participants with distal metastases alone. The participants with nodal recurrence underwent salvage neck dissection. There were therefore 50/56 patients with 2‐year DFS.

#### 3.1.1 Associations between post‐CRT DW‐MRI and PET/CT parameters

The descriptive statistics for the tumor and nodal ADC_mean_, ADC_min_, SUV_max_, SUV_max_ lesion: liver at 12‐week posttreatment imaging, together with interval changes from pretreatment imaging are shown in Table 3.

**TABLE 3:** Descriptive statistics of ADC_min_ (×10^−6^ mm^2^/s), ADC_mean_ (×10^−6^ mm^2^/s), SUV_max_ and SUV_max_: liver‐to‐target ratio values for node and tumor, measured at 12‐weeks posttreatment, and absolute interval change between pretreatment and 12‐weeks posttreatment values

	n	Mean	SD	LQ	Median	UQ
Node ADC_min_ at 12 weeks	52	1028.70	245.04	888.25	1030.00	1163.73
Tumor ADC_min_ at 12 weeks	48	1373.79	282.27	1181.00	1369.46	1583.78
Change in node ADC_min_ pre‐12 weeks	52	338.07	343.62	159.75	337.00	470.71
Change in tumor ADC_min_ pre‐12 weeks	48	732.17	333.37	461.62	780.00	971.00
Node ADC_mean_ at 12 weeks	52	1462.88	250.71	1326.14	1458.28	1573.10
Tumor ADC_mean_ at 12 weeks	48	1830.08	270.47	1614.87	1821.60	1984.39
Change in node ADC_mean_ pre‐12 weeks	52	520.74	322.15	261.54	513.90	713.33
Change in tumor ADC_mean_ pre‐12 weeks	48	926.01	294.51	745.32	921.63	1089.01

	n	Mean	SD	LQ	Median	UQ
Node SUV_max_ at 12 weeks	52	1.89	0.55	1.52	1.83	2.33
Tumor SUV_max_ at 12 weeks	48	3.00	0.88	2.32	2.96	3.62
Change in node SUV_max_ pre‐12 weeks	36	−7.11	4.43	−9.95	−6.39	−4.25
Change in tumor SUV_max_ pre‐12 weeks	30	−10.24	6.78	−13.73	−10.31	−5.15
Liver: node SUV_max_ at 12 weeks	52	0.72	0.20	0.56	0.71	0.88
Liver: tumor SUV_max_ at 12 weeks	48	1.17	0.35	0.91	1.09	1.36
Change in liver: node SUV_max_ pre‐12 weeks	36	−2.57	1.75	−3.58	−2.33	−1.75
Change in liver: tumor SUV_max_ pre‐12 weeks	30	−3.74	2.67	−5.01	−3.48	−1.65

Abbreviations: LQ, lower quartiles; UQ, upper quartiles.

**FIGURE 2:**
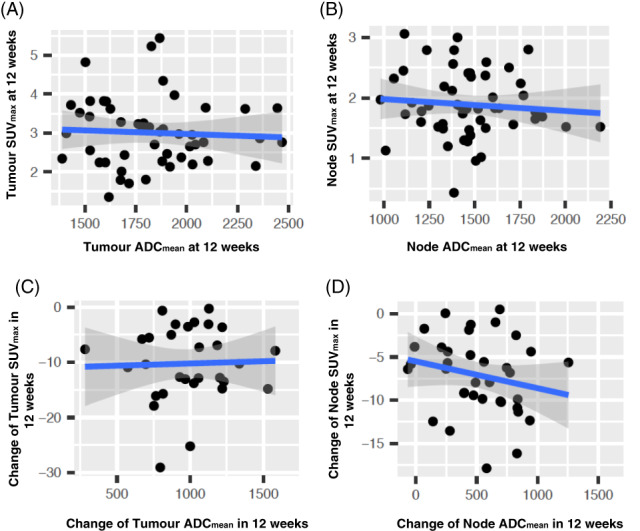
Scatter plots of SUV_max_ vs ADC_mean_ at 12‐weeks post CRT for (A) primary tumor and (B) lymph node as well as scatter plots for changes in SUV_max_ vs changes in ADC_mean_ from pretreatment to 12‐week posttreatment for (C) primary tumor and (D) lymph node. PET measures (*y*‐axes) and MRI measures (*x*‐axes). Line of best fit (blue) with 95% confidence intervals shown overlaid (grey)

**FIGURE 3:**
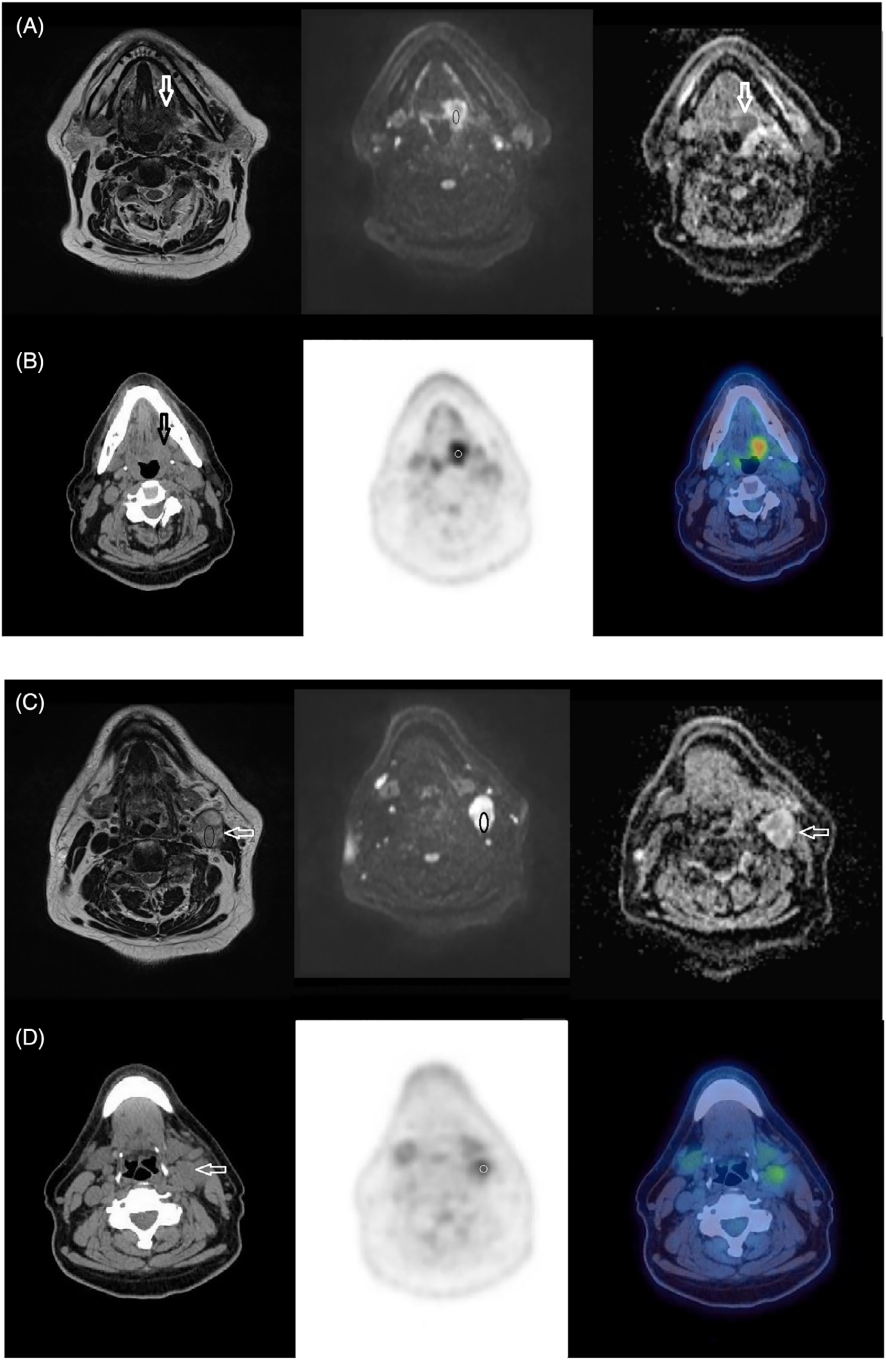
Pretreatment MRI and FDG PET/CT study in a 64‐year‐old male patient with T2N2b left oropharynx tumor. T2W, *b* = 800 DWI and ADC map (*b* = 100‐800) MRI images (A and C) and CT, PET, PET/CT fused images (B and D) indicating the left glosso‐tonsillar sulcus tumor (arrows in A and B) and left level 2 lymph node (arrows in C and D). The ROIs on the MRI study include areas of increased DWI signal corresponding to intermediate T2w signal in the cores of the primary and nodal tumor as indicated (ovals in A and C). The 6 mm VOI on the PET‐CT study is seen within the central portion of the primary and nodal tumor (circles in B and D)

**FIGURE 4:**
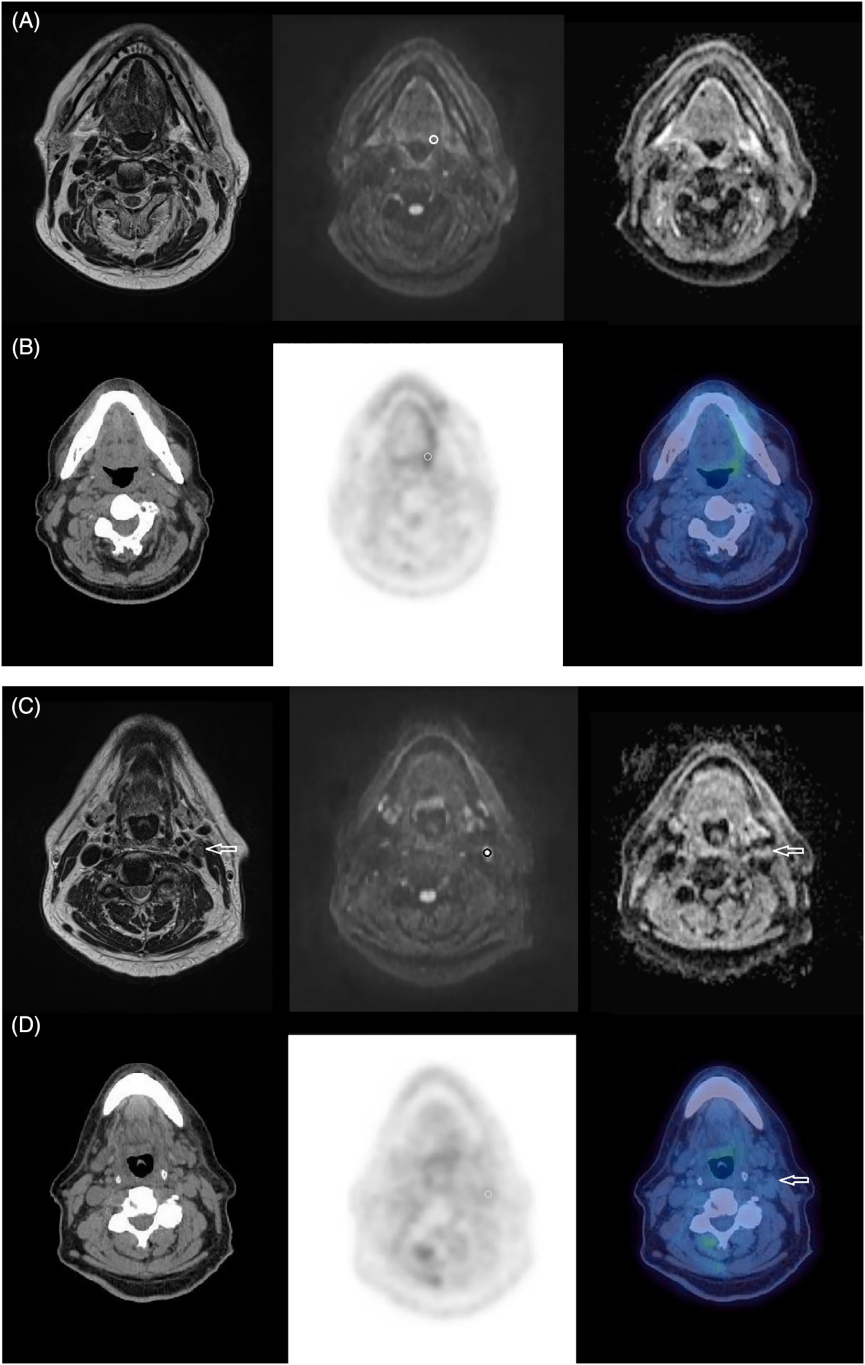
12‐week post‐chemo‐radiotherapy MRI and PET/CT study in the same patient. T2w, *b* = 800 DWI and ADC map (*b* = 100‐800) MRI images (A and C) and CT, PET, PET/CT fused image (B and D) indicating the site of the previous left glosso‐tonsillar sulcus tumor, which is now nonmeasurable and left level 2 lymph node, which has markedly reduced in size (arrows in C and D). The 6 mm ROIs on the MRI study are placed at the site of the previous primary tumor (circle in A) and at the residual nodal tumor as indicated (circle in B). The corresponding 6 mm VOI on the PET‐CT study is seen within the central portion of the primary and nodal tumor (circles in B and D)

No significant negative correlations were demonstrated between ADC_mean_ and SUV_max_ at the tumor and nodal sites on the 12‐week post‐CRT studies (*P* = .455‐.794; tau = −0.075‐0.25), or their interval changes from pretreatment to 12‐week posttreatment studies (*P* = .1‐.946; tau = −0.194‐0.044). Scatter plots of the correlation are demonstrated in Figure 2. There was also no correlation between any of the other DW‐MRI (ADC_min_) and ^18^F‐FDG PET/CT (SUV_max_ lesion: liver) parameters with respect to the 12‐week posttreatment studies or pretreatment to 12‐week posttreatment interval changes (Figures 3 and 4). Table 4 demonstrates the correlation of MRI parameters and PET/CT parameters, with Table 5 demonstrating the same correlation but restricted to patients with measurable disease (n = 19/52 lymph nodes) at 12‐weeks posttreatment.

**TABLE 4:** Correlation of MRI‐DWI parameters (ADC_min_ and ADC_mean_) against PET parameters (SUV_max_, SUV_max_: liver‐to‐target ratio) at 12 weeks, and absolute interval change between pretreatment and 12 weeks posttreatment values

MRI (X)	PET (Y)	n	tau	*P*‐value
Node ADC_mean_ at 12 weeks	Node SUV_max_ at 12 weeks	52	−0.061	.528
Node ADC_min_ at 12 weeks	Node SUV_max_ at 12 weeks	52	−0.025	.794
Tumor ADC_mean_ at 12 weeks	Tumor SUV_max_ at 12 weeks	48	−0.075	.455
Tumor ADC_min_ at 12 weeks	Tumor SUV_max_ at 12 weeks	48	−0.042	.676
Node ADC_mean_ at 12 weeks	SUV_max_ liver: node at 12 weeks	52	−0.027	.776
Node ADC_min_ at 12 weeks	SUV_max_ liver: node at 12 weeks	52	−0.034	.723
Tumor ADC_mean_ at 12 weeks	SUV_max_ liver: tumor at 12 weeks	48	−0.048	.639
Tumor ADC_min_ at 12 weeks	SUV_max_ liver: tumor at 12 weeks	48	−0.059	.551
Node ADC_mean_ change pre‐12 weeks	Node SUV_max_ change pre‐12 weeks	36	−0.194	.100
Node ADC_min_ change pre‐12 weeks	Node SUV_max_ change pre‐12 weeks	36	−0.101	.946
Tumor ADC_mean_ change pre‐12 weeks	Tumor SUV_max_ change pre‐12 weeks	30	0.030	.832
Tumor ADC_min_ change pre‐12 weeks	Tumor SUV_max_ change pre‐12 weeks	30	0.039	.762
Node ADC_mean_ change pre‐12 weeks	SUV_max_ liver: node change pre‐12 weeks	36	−0.175	.138
Node ADC_min_ change pre‐12 weeks	SUV_max_ liver: node change pre‐12 weeks	36	0.022	.860
Tumor ADC_mean_ change pre‐12 weeks	SUV_max_ liver: tumor pre‐12 weeks	30	0.025	.860
Tumor ADC_min_ change pre‐12 weeks	SUV_max_ liver: tumor change pre‐12 weeks	30	0.044	.735

**TABLE 5:** Correlation of MRI‐DWI parameters (ADC_min_ and ADC_mean_) against PET parameters (SUV_max_, SUV_max_ liver‐to‐target ratio) in patients with measurable nodal disease at 12 weeks, and absolute interval change between pretreatment and 12‐weeks post treatment values

MRI (X)	PET (Y)	n	tau	*P*‐value
Node ADC_mean_ at 12 weeks	Node SUV_max_ at 12 weeks	19	−0.035	.834
Node ADC_min_ at 12 weeks	Node SUV_max_ at 12 weeks	19	−0.199	.234
Node ADC_mean_ at 12 weeks	SUV_max_ liver: node at 12 weeks	19	0.006	1.000
Node ADC_min_ at 12 weeks	SUV_max_ liver: node at 12 weeks	19	−0.158	.368
Node ADC_mean_ change pre‐12 weeks	Node SUV_max_ change pre‐12 weeks	11	−0.236	.359
Node ADC_min_ change pre‐12 weeks	Node SUV_max_ change pre‐12 weeks	11	−0.055	.879
Node ADC_mean_ change pre‐12 weeks	SUV_max_ liver: node change pre‐12 weeks	11	−0.236	.359
Node ADC_min_ change pre‐12 weeks	SUV_max_ liver: node change pre‐12 weeks	11	−0.055	.879

#### 3.1.2 Prediction of 2 year DFS with post‐CRT DW‐MRI and PET/CT parameters

The comparison of ADC and SUV_max_ parameters in participants with and without 2 year DFS is demonstrated in Table 6. The primary tumor ADC_mean_ at 12‐weeks post‐CRT DW‐MRI (*P* = .03) and the interval change in nodal ADC_min_ from pretreatment to 12‐weeks post‐CRT DW‐MRI (*P* = .05) were associated with 2‐year DFS. None of the other ADC parameters and no SUV_max_ parameters were able to predict 2‐year DFS.

**TABLE 6:** Two‐year disease‐free survival and comparison of ADC and SUV_max_ parameters in participants with and without 2‐year disease‐free survival

Parameter	Total no. participants	No 2 year DFS (no. participants)	2 year DFS (no. participants)	*P* value (parameter when no 2 year DFS vs 2 year DFS)
Node ADC_min_ at 12 weeks	52	6	46	.06
Tumor ADC_min_ at 12 weeks	48	6	42	.89
Change in node ADC_min_ pre‐12 weeks	52	6	46	.05
Change in tumor ADC_min_ pre‐12 weeks	48	5	43	.75
Node ADC_mean_ at 12 weeks	52	6	46	.08
Tumor ADC_mean_ at 12 weeks	48	6	42	.03
Change in node ADC_mean_ pre‐12 weeks	52	6	46	.10
Change in tumor ADC_mean_ pre‐12 weeks	48	5	43	.88

Parameter	Total no. participants	No 2 year DFS (no. participants)	2 year DFS (no. participants)	*P* value (parameter when no 2 year DFS vs 2 year DFS)
Node SUV_max_ at 12 weeks	52	6	46	.48
Tumor SUV_max_ at 12 weeks	48	6	42	.21
Change in node SUV_max_ pre‐12 weeks	36	3	33	.92
Change in tumor SUV_max_ pre‐12 weeks	30	2	28	.86
Liver: node SUV_max_ at 12 weeks	52	6	46	.27
Liver: tumor at 12 weeks	48	6	42	.40
Change in liver: node SUV_max_ pre‐12 weeks	36	3	33	.82
Change in liver: tumor SUV_max_ pre‐12 weeks	30	2	28	.57

## 4 DISCUSSION

This study provides novel data comparing post‐CRT diffusion quantitative DW‐MRI and ^18^F‐FDG PET/CT parameters in HNSCC. No correlation was demonstrated between ADC_mean_ and SUV_max_ on 12‐week post‐CRT studies, or between the interval change in ADC_mean_ and SUV_max_ values from pretreatment to 12‐week post‐CRT studies in this HPV OPC dominant cohort with stage III and IV HNSCC. There was also no relationship when comparisons were extended to alternative posttreatment ^18^F‐FDG PET and MRI parameters (ADC_min_, SUV_max_ ratio of tumor to liver) or when only participants with measurable disease on posttreatment DW‐MRI studies were analyzed. Only the 12‐week post‐CRT primary tumor ADC_mean_ and interval change between pretreatment and 12‐week post‐CRT nodal ADC_min_ were predictive of 2 year DFS.

^18^F‐FDG PET/CT and DW‐MRI play an increasing role in the management of high stage HNSCC, and have both been used to provide prognostic biomarkers following CRT treatment. SUV indicates tumor metabolism and ADC reflects microscopic features such as tumor cellularity. ADC may also represent a surrogate marker for hypoxia as indicated by ^18^F‐FMISO PET/CT.^37^ While some studies have focused on the role of pretreatment SUV_max_ ^34^, ^35^, ^38^, ^39^, ^40^, ^41^, ^42^, ^43^, ^44^, ^45^ and ADC in assisting the early prediction of treatment failure,^6^, ^20^, ^21^, ^23^, ^34^, ^36^, ^37^, ^38^, ^39^, ^40^, ^41^, ^42^, ^43^, ^44^, ^45^, ^46^, ^47^, ^48^, ^49^, ^50^ a posttreatment assessment of SUV_max_ and ADC values has proved most useful to date and was the focus of this study.

Posttreatment ^18^F‐FDG PET/CT is an established technique for the evaluation of post‐CRT advanced HNSCC.^2^, ^9^, ^10^, ^11^, ^12^ Since treatment‐induced inflammation in the very early posttreatment period may lead to false positive studies,^16^ ^18^F‐FDG PET/CT is usually delayed until 12 weeks following the completion of CRT in order to increase the specificity.^2^, ^17^, ^18^ Semiquantitative analysis demonstrating increased posttreatment SUV_max_ values and lack of significant reduction in SUV_max_ values within loco‐regional tumor has been shown to indicate treatment failure.^13^, ^14^, ^15^, ^16^, ^17^, ^18^, ^29^, ^33^ Similarly, quantitative DW‐MRI has been investigated in the posttreatment setting, with a higher ADC, or a greater interval increase from pre‐ to intra‐ or posttreatment ADC,^19^, ^20^, ^21^, ^22^, ^23^, ^24^ being associated with loco‐regional treatment success.

It would therefore be useful to establish whether the posttreatment DW‐MRI and ^18^F‐FDG PET/CT‐based parameters are correlated with each other. While treatment failure is associated with interval changes in both posttreatment SUV_max_ and ADC, they may still have a complementary role in evaluating treatment response if they are demonstrated to be independent variables.^34^, ^35^ This would inform on appropriate early posttreatment protocols and the applicability of new technologies such as PET‐MRI, which would measure both parameters simultaneously.

Previous reports comparing SUV and ADC parameters in HNSCC are restricted to the pretreatment scenario and these have demonstrated disparate results. Choi et al (n = 31)^30^ and Nakajo et al (n = 26)^34^ reported significant negative correlations between pretreatment ADC and SUV. It was argued that the glycolytic activity evaluated with ^18^F‐FDG PET‐CT is therefore significantly related with the microstructural environment evaluated by DW‐MRI in patients with HNSCC. In contrast, Varoqaux et al (n = 33)^36^ and Freuhwald‐Wallamar et al (n = 46)^31^ showed no statistical relationship, and hence identified pretreatment SUV and ADC as potentially independent biomarkers in HNSCC. One study has compared quantitative DW‐MRI and ^18^F‐FDG PET/CT parameters in the posttreatment setting at another tumor site, with ADC_min_ and SUV_max_ found not to be significantly correlated in recurrent cervical (gynecological) cancer.^51^

Alternative ^18^F‐FDG PET/CT and ADC parameters were also evaluated. First, although SUV is the more commonly used parameter in the assessment of HNSCC treatment response,^13^, ^14^, ^15^, ^16^, ^17^, ^18^ the role of absolute SUV values in the posttreatment evaluation of HNSCC with ^18^F‐FDG PET/CT has been questioned.^10^, ^52^ As an alternative, ^18^F‐FDG uptake may be measured relative to normal tissue/background tissue, and quantitative interpretative criteria such as the Porceddu, Hopkins, and Deauville scoring systems are based on this approach.^53^, ^54^, ^55^ Zhong et al evaluated these scoring systems and found that they demonstrated high specificity, PPV, and NPV.^56^ Tumor uptake exceeding liver tracer uptake is indicative of disease in all these criteria, and hence we decided to include tumor SUV to liver ratio as another ^18^F‐FDG PET/CT parameter. Some previous studies have demonstrated total lesional glycolysis (TLG) to be a superior predictor of HNSCC treatment outcomes^57^; however, this requires an assessment of metabolic tumor volume, which was not possible in many of the posttreatment cases, where there was no definable FDG uptake and a standardized small VOI was placed. Second, with respect to the DW‐MRI parameters, both ADC_mean_ and ADC_min_ were evaluated in this study since each has previously been applied to previous comparisons of quantitative DW‐MRI with ^18^F‐FDG PET/CT on pretreatment imaging.^35^, ^36^

It is of note that while selected ADC parameters were able to predict treatment outcomes in this study, none of the SUV_max_ parameters proved prognostically useful in this study. Some previous studies have shown that posttreatment SUV_max_ is a less accurate predictor of outcome in HPV OPC cohorts.^58^, ^59^ In addition, the unexpectedly high rate of HPV‐OPC participant recruitment also resulted in low rate of treatment failure such that it was suboptimally powered for the comparison of quantitative DW‐MRI and ^18^F‐FDG PET/CT parameters with 2‐year DFS.

There are potential shortcomings with the study methodology. First, there are greater challenges with the accurate placement and measurement of ADC and SUV in the posttreatment setting. For instance, there is a reduction in conspicuity of any anatomical tumor target on MRI and the qualitative assessment of the tumor site may not detect FDG tracer greater than background on ^18^F‐FDG PET/CT. This resulted in there being only one third of nodal sites and no primary tumor sites which demonstrated a clear DWI focus to guide the ROI placement on the 12‐week posttreatment DW‐MRI studies. In addition, the DW‐MRI and 18F‐PET‐CT measurements should ideally have been colocalized, but no fusion of the MRI and 18F‐PET/CT data sets was feasible. Second, we do not present interobserver agreement statistics as part of this study and this would be particularly pertinent to the posttreatment analysis. Varoquaz et al^36^ has previously evaluated reproducibility of pretreatment ADC and SUV measurements with ICC > 0.9 for both. Third, the HPV‐OPC dominant cohort is a potential confounding factor in this study with its unique histopathological characteristics^60^ and differing tumor metabolism.^61^ It is recognized that pretreatment ADC values are lower and over a wider range in HPV OPC,^62^, ^63^ which may influence posttreatment ADC values or their interval change.

## 5 CONCLUSION

We provide the first direct comparison of posttreatment DW‐MRI and ^18^F‐FDG PET/CT variables and their interval change from pretreatment values in patients with stage III and IV HNSCC. There was no significant negative linear relationship between ADC and SUV values on 12‐week post‐CRT studies or their interval changes in this HPV OPC dominant cohort. None of the SUV_max_ parameters and only selected ADC parameters were associated with 2‐year disease‐free outcome. Since both the relationship between ADC and SUV values and the prognosis is influenced by HPV OPC status, further studies should focus on HPV negative HNSCC to determine whether DW‐MRI and ^18^F‐FDG PET/CT provide independent biomarkers in the post‐CRT setting.

## AUTHOR CONTRIBUTIONS

All authors had full access to the data in the study and take responsibility for the integrity of the data and the accuracy of the data analysis. *Conceptualization*, SC, VG; *Methodology*, SC, VG, GC, TS; *Investigation*, SC, CS, MA; *Formal Analysis*, SC, JD; *Data Curation*, MA, SC; *Writing—Original Draft*, SC; *Writing—Review & Editing*, SC, VG, GC, TS, IP; *Visualization*, JD, SC, CS; *Supervision*, SC, VG; *Project Administration*, SC; *Funding Acquisition*, SC.

## CONFLICT OF INTEREST

The authors have stated explicitly that there are no conflicts of interest in connection with this article.

## ETHICAL STATEMENT

Institutional approval from the Research Ethics Committee (REC reference 13/LO/1876) and informed consent were obtained from all participants. The study conforms to recognized standards of the Declaration of Helsinki.

## Data Availability

The data that support the findings of this study are available from the corresponding author upon reasonable request.
